# Ileocecal resection for massive rectal bleeding due to *Yersinia enterocolitica*: a case report and review of the literature

**DOI:** 10.1186/s13256-015-0786-2

**Published:** 2016-01-19

**Authors:** Ilham Azghari, Aicha Bargach, Nabil Moatassim Billah, Mohamed Amine Essaoudi, Ahmed Jahid, Nawal Kabbaj

**Affiliations:** 1 EFD-Hepatogastroenterology, Ibn Sina Hospital, Rabat, Morocco; 2 Radiology Unit, Ibn Sina Hospital, Rabat, Morocco; 3 Anatomopathology Unit, Ibn Sina Hospital, Rabat, Morocco

**Keywords:** Ileocecal resection, Pseudotumoral aspect, Rectal bleeding, *Yersinia enterocolitica*

## Abstract

**Introduction:**

Massive gastrointestinal bleeding is an emergency that can sometimes require immediate surgery. We report the first case, to the best of our knowledge, of massive rectal bleeding due to *Yersinia enterocolitica*, requiring ileocecal resection.

**Case presentation:**

A 41-year-old North African woman was admitted to our emergency department for massive rectal bleeding. She had a history of an iron deficiency anemia of unknown cause, and diarrhea 2 months before the admission. On admission to our emergency unit, she was in a state of hemodynamic collapse. An examination showed discolored conjunctivas, massive rectal bleeding with clots and no abdominal pain. The first medical treatment included the use of noradrenaline. An upper gastrointestinal endoscopy was performed and did not show any lesions. Computed tomography of her abdomen showed significant and hypervascular wall thickening of her terminal ileum suggestive of a tumor. Because her massive rectal bleeding worsened and her collapse persisted, an exploratory laparotomy and ileocecal resection were immediately performed on the patient. Histopathological analysis showed enteritis caused by *Yersinia enterocolitica*. Her outcome was favorable.

**Conclusion:**

Enteritis due to *Yersinia enterocolitica* can take a pseudotumoral form and mislead the diagnosis of gastrointestinal bleeding.

## Introduction

*Yersinia enterocolitica* is a bacterium responsible for enteritis. Its symptoms are usually abdominal pain, fever, vomiting and diarrhea. Several case reports present unusual manifestations of *Yersinia* infections. Pseudotumoral forms and bowel perforations were reported. We here report a case of massive rectal bleeding resulting in collapse due to *Yersinia enterocolitica.*

## Case presentation

A 41-year-old North African woman with a history of chronic anemia with iron supplementation intake presented to our emergency department. She had massive rectal bleeding.

She had an iron deficiency anemia. She did not complete the investigations so the cause was not yet diagnosed. She had no chronic bleeding, neither gynecologic nor digestive. She was treated by oral iron with no regular follow up. She had no past surgical interventions, and no other medical condition. She had no contact with animals. There were no medical conditions in her family, no cancers and no current infections. No one in her family or neighborhood was diagnosed with tuberculosis.

In our admission unit, her hemodynamic parameters showed that she was in shock: her blood pressure was 80/40 mmHg and her pulse was up to 120 beats per minute. She was pale, her conjunctivas were discolored and her limbs were cold. While examining her, we found profuse rectal bleeding with clots. A quick abdominal and pelvis examination including proctologic examination appeared normal. She was transferred to our intensive care unit and monitored.

Noradrenaline was administered because the crystalloid fluids and the transfusion were not sufficient to compensate for her blood loss. Several blood tests were conducted, including blood count and clotting tests. Her hemoglobin was 8 g/dl and hematocrit was 29 %. The rest of the formula and the clotting tests returned normal results as shown in Table 1.

**Table 1:** Results of biological tests on the day of admission

Hgb	MCV	MCHC	Hematocrit	WBC	Platelets	PR	Urea	Creatinin	Sodium	Potassium
8 g/dl	81	32	29 %	5660	160,000	87 %	0.3 g/l	12 mg/l	135 mEq/l	4.1 mEq/l

*Hgb* hemoglobin, *MCHC* mean corpuscular hemoglobin concentration, *MCV* mean corpuscular volume, *PR* prothrombin ratio, *WBC* white blood cells

**Fig. 1:**
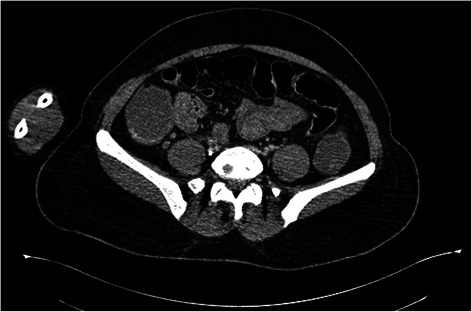
Abdominal computed tomography of the abdomen showing an irregular wall thickening

**Fig. 2:**
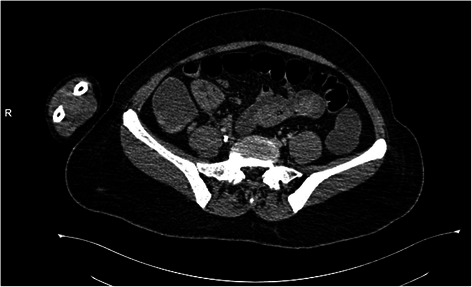
Abdominal computed tomography of the abdomen showing an ileocecal wall thickening

**Fig. 3:**
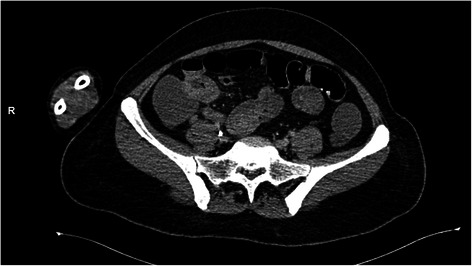
Abdominal computed tomography of the abdomen showing regional adenomegalies

**Fig. 4:**
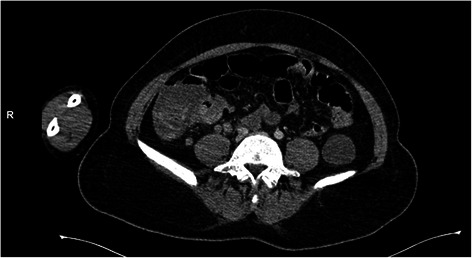
Abdominal computed tomography of the abdomen showing an infiltrative aspect and regional nodes

She first underwent an esogastroduodenoscopy, to identify the source of the bleeding. No abnormality was detected in the said procedure: the esophageal and gastric mucosa were normal, no ulcerations or traces of blood were found, and the duodenal mucosa was also explored and appeared normal. A colonoscopy could not be carried out because of the profuse rectal bleeding. She underwent a computed tomography (CT) of her abdomen which revealed a wall thickening in the terminal ileum with hypervascularization and regional adenomegalies (Figs. 1, 2, 3 and 4). This aspect suggested either a tumor or an inflammatory bowel disease.

Because her bleeding and her hemodynamic collapse persisted, it was decided to proceed with surgery with the aim of diagnosis and treatment. She underwent exploratory laparotomy immediately. It confirmed the wall thickening in her ileocecal junction. Her appendix was inflamed. An ileocecal resection was performed with an ileocolic anastomosis, removing 10 cm from her ileum. The specimen was sent for histological examination. Gross examination showed a round ulcer 5 cm from the ileal end, next to another ulcerative lesion; eight lymphatic nodes were found. The blood vessel under the deep ulcer was the origin of the profuse bleeding.

**Fig. 5:**
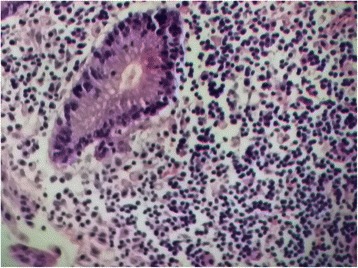
Histopathological examination of the colic mucosa showing an inflammatory aspect with regular glands

**Fig. 6:**
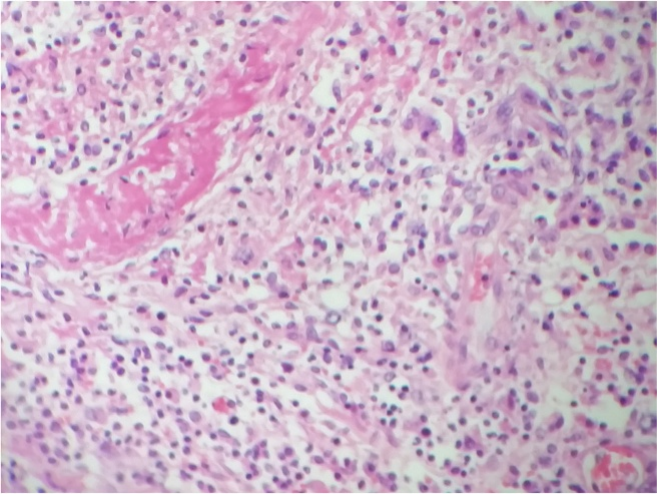
Histopathological examination of the ileocecal tissue showing inflammation and microabcesses

**Fig. 7:**
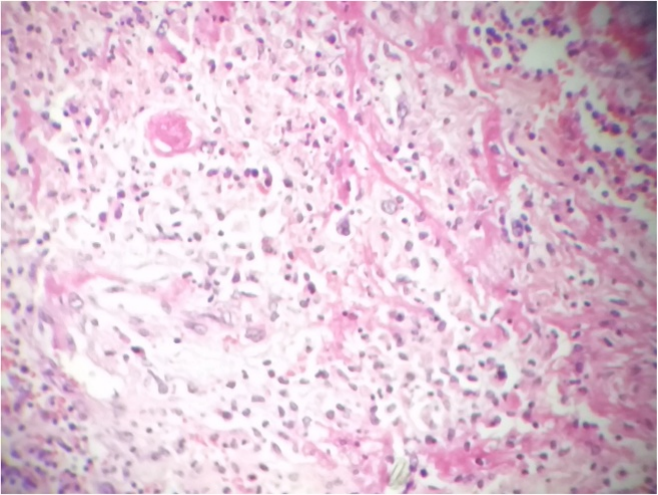
Histopathological examination of the ileocecal tissue showing marks of blood

**Fig. 8:**
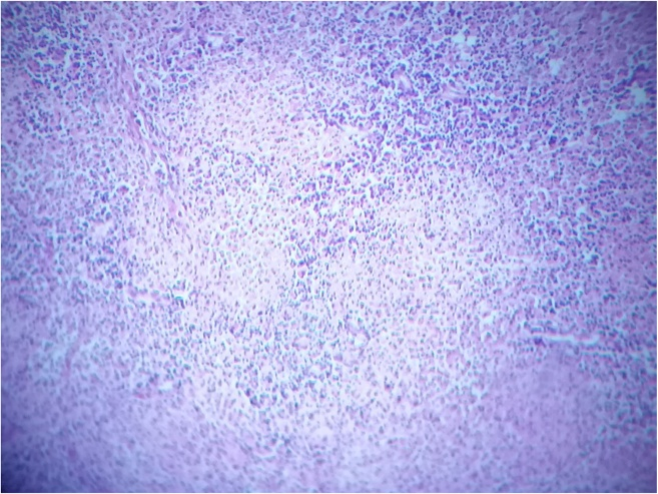
Histopathological examination of the adenomegalies showing a necrosis

The histopathological examination showed an inflammatory ulcerative reaction with microabscesses (Figs. 5, 6 and 7). A granulomatous necrotic reaction was revealed in the examination of all eight nodes (Fig. 8). The pathologist’s report concluded with diagnosis of an infection due to *Yersinia enterocolitica*.

Her immediate postoperative outcome was favorable, the bleeding stopped, and her hemodynamic parameters remained stable. She had a normal transit on the fifth day after the intervention. After receiving the pathologist’s report, it was decided to treat her with fluoroquinolones 500 mg twice daily without discontinuing the iron supplementation, and a clinical control was scheduled. At the control 3 weeks later, she had no clinical symptoms, her diarrhea and bleeding had ended, and signs of anemia were diminishing. Antibiotherapy was discontinued.

## Discussion

*Yersinia* is a genus of Gram-negative aerobic facultative bacilli. *Yersinia enterocolitica* is one of the species in this genus that can cause human diseases. The other two are *Yersinia pseudotuberculosis* and *Yersinia pestis* [1]. It is found in milk, pork and other domestic animals. The transmission can happen through consumption of contaminated food or water [2]. It is more frequent in the pediatric population (less than 10-years old) [3].

The clinical manifestations of a *Yersinia enterocolitica* infection in humans include fever, abdominal pain, diarrhea and vomiting. In a study reported by Saebø *et al*., which registered the clinical aspects in patients with yersiniosis for a duration of 9 years, predominant symptoms were diarrhea, abdominal pain, vomiting and weight loss [4]. The appendix is involved in 40 % of cases according to Vantrappen *et al*. [5]. Its tropism for the lymphoid tissue explains the pseudo-appendicular form and the mesenteric lymphadenitis [6]. In fact, in a reported outbreak of *Yersinia enterocolitica* infection associated with contaminated chocolate milk, half of the affected patients underwent laparotomy for suspected acute appendicitis [7]. Extraintestinal manifestations can occur. Cases of septic arthritis have been reported [8]. Patients with immune depression, diabetes, iron overload, cirrhosis or hepatic disease are particularly susceptible to disseminated Y*ersinia enterocolitica* infection [9]. Hepatic abscesses, septicemia and encephalitis have been reported in such cases [10]. Bacteremia has also been reported in babies [11]. The infection can last several months. The evolution that had been noticed indicates that yersiniosis is a chronic state [4].

Radiological aspects include terminal ileitis over a distance of 10 to 20 cm. Irregular or nodular mucosal pattern and pictures suggestive of ulcerations are the most frequent radiological signs. Signs of colitis and aphthoid ulcers are noticed in endoscopy in some cases [5]. In fact, Naddei *et al*. report a case of yersiniosis mimicking Crohn’s disease in an 11-year-old boy with recurrent abdominal pain [12]: “Ileo-colonoscopy showed the presence of granularities and aphthous ulcers at the terminal ileum” [12]. Homewood *et al*. reported a case of fulminating ileitis in a 20-year-old woman with abdominal pain and diarrhea that turned out to be a *Yersinia pseudotuberculosis* infection [13]. The CT showed a thickening in the terminal ileum and their surgical exploration found ileal ulcers. The woman was subsequently diagnosed with Crohn’s disease. In fact, *Yersinia* infection was reported to be associated in some cases with intestinal bowel disease (IBD) [13, 14]. This was also noticed through the long-term follow up of patients with initial yersiniosis [4, 15].

*Yersinia* infection often manifests with gastrointestinal ulcers, especially in the terminal ileum; perianal and colonic ulcers have also been reported [16]. The presence of gastrointestinal ulcers can cause minor digestive bleeding leading to iron deficiency anemia but very rarely a massive bleed and a collapse as was the case for our patient.

*Yersinia* infection can also take a pseudotumoral form on radiological examination. A case of pseudotumoral form due to *Yersinia pseudotuberculosis* that required a hemicolectomy was reported [17]: a 56-year-old man admitted for abdominal pain, vomiting and distension. Clinical examination was suggestive of intestinal obstruction. A CT scan showed wall thickening in the cecum and the terminal ileum. The patient underwent exploratory laparotomy with right hemicolectomy. The diagnosis of *Yersinia pseudotuberculosis* enteritis was revealed through the histopathological examination [17].

Ileal perforation due to *Yersinia enterocolitica* has also been reported [18]. In this case, the patient needed surgery for pelviperitonitis due to an intestinal perforation. Necrotic-ulcerative ileitis with adenomesenteritis from *Yersinia enterocolitica* was found. The progression was favorable after intestinal resection and antibiotic therapy [18]. Only one case of small bowel gangrene due to *Yersinia enterocolitica* was reported in the literature [19]. There is also one reported instance of gangrene due to *Yersinia pseudotuberculosis* [20].

Diagnosis of *Yersinia enterocolitica* infection is usually suspected from the clinical presentation. It is then confirmed by blood and stool cultures, serological tests and histopathological examination [6, 11, 20, 21]. The best antibiotic treatment seems to be fluoroquinolones, alone or in combination, or third-generation cephalosporins [9].

In our case, the patient presented diarrhea for 2 months followed by an acute episode of massive rectal bleeding, with hemodynamic collapse. Proctologic examination did not show any anal lesions. In this case, the most frequent etiologies are angiodysplasia and diverticulosis, but massive rectal bleeding can also be caused by an upper gastrointestinal lesion: gastric or duodenal ulcer, esophageal varices, tumors or angiodysplasia. An upper endoscopy of our patient showed a normal esogastroduodenal mucosa. It was not possible to perform a colonoscopy given the urge of obtaining a diagnosis for her. Therefore, it was decided to proceed with an abdominal CT with an injection of contrast material. The abdominal CT revealed ileocecal pathology: wall thickening in her terminal ileum with hypervascularization and regional adenomegalies.

An IBD, particularly Crohn’s disease, was suspected. Reasons for this were her young age, gender (female), history of diarrhea in the preceding few months and past chronic iron deficiency anemia, digestive bleeding and the ileocecal localization. Deep ulcers would have been the origin of the massive bleeding. This happens in 5 % of IBD [22].

The presence of a tumor such as adenocarcinoma could not be ruled out. Grelic and colic tumors can be responsible for 11 to 20 % of low digestive bleeding [23]. The fast progression of the symptomatology in our patient’s case, her age, and the absence of familial neoplasia made this less likely. However, the hypervascular characteristic and the regional nodes suggested a carcinoid tumor. This latter can be revealed through a complication such as digestive bleeding. The presence of diarrhea was also an argument, although the carcinoid syndrome was not complete.

Her massive bleeding causing a hemodynamic collapse made the infectious origin less probable. Nevertheless, ileocecal tuberculosis could not be ruled out because of the frequency of tuberculosis in the country. Concerning other infectious causes, *Yersinia*, *Campylobacter* and *Salmonella* are the ones most frequently associated with ileal involvement, and pseudotumoral forms have been reported especially in *Yersinia enterocolitica* and *Yersinia pseudotuberculosis*, but no case of massive bleeding was reported.

At the surgical exploration step, gross examination of the tissue revealed ulcers and it confirmed the wall thickening of her terminal ileum. This kept the diagnosis discussion focused on the same path. A histopathological examination unexpectedly revealed signs of *Yersinia enterocolitica* enteritis. Her clinical evolution was favorable. Her bleeding and diarrhea stopped and her hemodynamic state remained stable. Her medium-term postoperative evolution was favorable as was her long-term follow up.

*Yersinia enterocolitica* infection can heal spontaneously with no complications. However, through this case report and the literature review, we see that some of its manifestations can be potentially fatal. This diagnosis has to be suspected whenever an acute abdominal syndrome is unexplained. Endoscopy is often performed to rule out other diagnoses. This allows for biopsy which helps to confirm the diagnosis. Microbacteriological tests can be helpful when they actively identify *Yersinia enterocolitica*. The treatment is generally based on antibiotic therapy but some cases need surgical intervention. The practical management of massive rectal bleeding due to *Yersinia enterocolitica* requires hemodynamic monitoring first. Surgery is needed to stop the bleeding if the latter is not controlled adequately.

## Conclusions

*Yersinia enterocolitica* is a species of *Yersinia* responsible for human disease. Abdominal pain, fever and diarrhea are noted in the most frequent clinical presentations of this infection. However, it can be responsible for pseudoappendicitis syndrome, terminal ileitis and wall thickening. Also, as it was in our case, it can cause significant digestive bleeding with an ulcerative and pseudotumoral form and regional nodes that can mislead the diagnosis. This case report is particularly relevant to the gastroenterological field, emergency medicine, surgery and the study of infectious diseases. Enteritis due to *Yersinia enterocolitica* or *Yersinia* in general is an etiology to keep in mind even if digestive symptoms mimic a surgical emergency. Through its ulcerative presentation, *Yersinia* infection can be the origin of digestive bleeding. Whenever it is possible, endoscopy with bacteriological and histopathological analysis can confirm the diagnosis, and radiology and surgery can also be helpful.

## Consent

Written informed consent was obtained from the patient for publication of this case report and accompanying images. A copy of the written consent is available for review by the Editor-in-Chief of this journal.

## Abbreviations

CT: computed tomography
IBD: intestinal bowel disease
